# Effectiveness and User Experience of a Smoking Cessation Chatbot: Mixed Methods Study Comparing Motivational Interviewing and Confrontational Counseling

**DOI:** 10.2196/53134

**Published:** 2024-08-06

**Authors:** Linwei He, Erkan Basar, Emiel Krahmer, Reinout Wiers, Marjolijn Antheunis

**Affiliations:** 1 Department of Communication and Cognition Tilburg School of Humanities and Digital Sciences Tilburg University Tilburg Netherlands; 2 Behavioral Science Institute Radboud University Nijmegen Netherlands; 3 Addiction Development and Psychopathology (ADAPT)-lab Department of Psychology and Centre for Urban Mental Health University of Amsterdam Amsterdam Netherlands

**Keywords:** chatbot, smoking cessation, counseling, motivational interviewing, confrontational counseling, user experience, engagement

## Abstract

**Background:**

Cigarette smoking poses a major public health risk. Chatbots may serve as an accessible and useful tool to promote cessation due to their high accessibility and potential in facilitating long-term personalized interactions. To increase effectiveness and acceptability, there remains a need to identify and evaluate counseling strategies for these chatbots, an aspect that has not been comprehensively addressed in previous research.

**Objective:**

This study aims to identify effective counseling strategies for such chatbots to support smoking cessation. In addition, we sought to gain insights into smokers’ expectations of and experiences with the chatbot.

**Methods:**

This mixed methods study incorporated a web-based experiment and semistructured interviews. Smokers (N=229) interacted with either a motivational interviewing (MI)–style (n=112, 48.9%) or a confrontational counseling–style (n=117, 51.1%) chatbot. Both cessation-related (ie, intention to quit and self-efficacy) and user experience–related outcomes (ie, engagement, therapeutic alliance, perceived empathy, and interaction satisfaction) were assessed. Semistructured interviews were conducted with 16 participants, 8 (50%) from each condition, and data were analyzed using thematic analysis.

**Results:**

Results from a multivariate ANOVA showed that participants had a significantly higher overall rating for the MI (vs confrontational counseling) chatbot. Follow-up discriminant analysis revealed that the better perception of the MI chatbot was mostly explained by the user experience–related outcomes, with cessation-related outcomes playing a lesser role. Exploratory analyses indicated that smokers in both conditions reported increased intention to quit and self-efficacy after the chatbot interaction. Interview findings illustrated several constructs (eg, affective attitude and engagement) explaining people’s previous expectations and timely and retrospective experience with the chatbot.

**Conclusions:**

The results confirmed that chatbots are a promising tool in motivating smoking cessation and the use of MI can improve user experience. We did not find extra support for MI to motivate cessation and have discussed possible reasons. Smokers expressed both relational and instrumental needs in the quitting process. Implications for future research and practice are discussed.

## Introduction

### Background

Cigarette smoking is one of the most preventable causes of premature death and illness [1], and supporting smokers in their attempts to quit is a public health priority. While the use of cessation support (eg, pharmacological treatment and behavioral support) can effectively increase the successful quitting rate [2], such services are facing substantial staff and resource shortages [3,4], which have been aggravated by the COVID-19 pandemic. Moreover, people tend to express resistance to using professional support when it comes to quitting smoking [5]. To combat these challenges, digital tools that can complement traditional cessation services have become increasingly popular. Conversational agents, or chatbots (ie, computer programs that use artificial intelligence to simulate a conversation with users through natural language [6]), are one of the recent innovations that bring several benefits: accessibility, scalability, personalization, and synchronicity, to name a few [7]. These opportunities have sparked interest to test the use of chatbots in health care settings, and initial effectiveness has been found in various domains, such as healthy lifestyle promotion [8], sexual health [9], mental health [10], and smoking cessation [11]. However, it is still unclear when and how chatbots are acceptable and effective. Some studies in human-computer interaction have shown that the positive effect of chatbots tends to decrease over time [12], and a few barriers that hinder human-chatbot interaction have been identified, including a lack of engagement caused by counseling styles that users do not prefer [13]. As long-term support is essential for smoking cessation, which usually requires sustained effort, and in view of the one-to-one nature of chatbots that allows for individualized conversation, it is important to find the optimal counseling style to overcome potential resistance to effectively motivate smoking cessation.

Within the field of individualized smoking cessation counseling, a chatbot can use various counseling styles, such as confrontational counseling (CC) and motivational interviewing (MI). CC originated in alcoholism treatment and emphasizes a hard-hitting and directive style intended to break through patients’ defense mechanisms [14]. CC counselors are trained to confront patients with the consequences of their unhealthy behaviors to counter self-exempting beliefs and use direct, unsolicited advice to increase patients’ risk perception [15]. In the field of smoking cessation, CC techniques include direct education about the risks associated with smoking, challenging smokers’ minimization or denial of the problem, and urging for abstinence by providing a quitting plan [16-18]. A typical example situation is the counselor coercing the client to *face up to reality* by emphasizing their problematic smoking behavior and the associated risks [14]. However, this does not necessarily entail an aggressive approach but rather attempts to raise clients’ awareness of the likely severe consequences of their smoking behavior [19]. Indeed, contrary to the common assumption that confrontation leads to denial [15], CC has been found to be predictive of higher patient involvement and higher long-term abstinence [20,21]. For several decades, CC has been regarded as the only way to get patients to listen [14], and there is positive evidence of health care providers using directive confrontational tactics to advise smokers to quit [22]. However, recent research suggests that the effectiveness and acceptability of CC depends on the counselor’s legitimacy and a rather empathetic manner [23], whereas resistance may escalate if the client feels threatened by the confrontational tone [24].

In response to the potential resistance, an alternative approach has been developed where patients’ autonomy and intrinsic motivation are highlighted [14,25]. Miller and Rollnick [25] introduced MI, a nonconfrontational counseling style for eliciting behavior change by helping clients explore and resolve ambivalence. The strategies of MI are more subtle than coercive, prioritizing encouragement over contention, with the ultimate goal of eliciting the client’s intrinsic motivation rather than imposing or desiring behavior change [25,26]. MI counselors use the principles of expressing empathy, avoiding arguing and confrontation, and supporting the individual’s self-efficacy. A typical example is “What you choose to do is up to you. My role is to help you figure out what you want to do and support you in accomplishing your goals” [27]. In the context of smoking cessation, MI counselors use techniques such as asking open-ended questions to elicit personally relevant reasons to quit, reflecting on the client’s words to encourage disclosure, affirming the client’s expressions toward quitting, and summarizing to help the client have a better overview of their thoughts [25,28]. The MI counselor assists the client in realizing the discrepancy between their values and their behaviors, thereby eliciting intrinsic motivation to resolve this discrepancy by changing their behavior [29,30].

Over the past decades, a rich amount of research on the effects of MI and CC on smoking cessation and other health behaviors has spawned, yielding mixed and inconclusive results. While CC has been found effective in increasing smokers’ risk perceptions and, in some cases, led to smoking cessation in the long run [20,31], there is also evidence suggesting that CC may lead to defense mechanisms among the clients (eg, denial and dismissal of the information) [32]. Similar inconclusiveness has been observed regarding the effectiveness of MI; while successful with other behavior domains (eg, alcohol use and physical exercise), the treatment tends to have weaker effects on smoking cessation [33,34]. Despite the mixed evidence regarding the 2 styles, modern addiction treatment research believes that MI is more likely to be successful than CC [14]. In general, MI-style interventions have been found to motivate a larger proportion of individuals than CC-style interventions, making them a more applicable and efficacious approach [14,35]. Moreover, the effectiveness of CC depends on the legitimacy and authority of the counselor [36]. In the context of chatbot-delivered interventions, CC might not be as effective as chatbots are usually not regarded as a medical authority [37]. On a broader note, it has been suggested that people prefer chatbots that offer emotional support (ie, MI) more than chatbots that predominantly offer informational support (ie, CC) [38]. In summary, MI seems to be a better approach for a smoking cessation chatbot.

### Objectives

However, it remains unclear whether the hypothesized advantage of MI over CC can be experimentally sustained in a chatbot setting. To date, only a few studies in human-delivered counseling have directly compared the 2 styles and found mixed results such that MI (vs CC) did not significantly reduce drinking and drug use but yielded less resistance and higher client satisfaction [14,18]. Moreover, no study has explicitly compared the 2 styles in a chatbot setting, and it is yet unknown whether the effect of MI is also applicable in automated settings. Human-computer interaction research in health care has emphasized the chatbots’ relational strategies to improve their effectiveness and acceptability [39,40], which are keenly matched to the principles of MI [25]. However, despite the theoretical advantages, the empirical evidence of automated MI is inconclusive [41]. Such insignificance of MI could be explained by the methodological disparities in the line of research: the comparison groups varied considerably, and the robustness of MI cannot be concluded. In a recent review of technology-delivered MI interventions [41], various studies compared MI with other types of interventions (eg, self-help booklet and assessment only) but not with an alternative counseling style (eg, CC). Therefore, the effectiveness of MI as a chatbot counseling style cannot be ascertained. In fact, regardless of the therapeutic counseling style, people might find a chatbot acceptable and even persuasive when presented nonintrusively [42], which, therefore, shadows the effect of chatbot counseling styles. Indeed, in an earlier study [43], an MI-style chatbot was compared with a neutral-style chatbot in motivating smokers to quit, expecting MI to generate a higher intention to quit and better therapeutic experience among smokers. However, no significant difference between the MI style and the neutral style was found. To follow up on previous research and further understand the role of the chatbot and better ascertain the usefulness of MI as a chatbot counseling style, this study compared the MI and CC therapeutic counseling styles in a chatbot setting. To sum up, this study hypothesized that an MI-style chatbot, compared to a CC-style chatbot, results in higher self-efficacy, stronger motivation to quit, more engagement, a stronger therapeutic alliance, more perceived empathy, and higher interaction satisfaction.

Moreover, to have a more comprehensive picture of whether and how an MI (vs CC) chatbot works and understand how users experience the chatbot interaction, we explored users’ perspectives on the chatbots through semistructured interviews. Specifically, we examined the following aspects: (1) What is the overall user experience with the smoking cessation chatbots? (2) How does the chatbot influence smokers’ perceptions of smoking and intention to quit? (3) How can we improve the chatbots for future use?

## Methods

### Study Design

This mixed methods study combined a quantitative part using a web-based experiment and a qualitative part of semistructured interviews. For the experiment, participants were randomly assigned to interact with a chatbot using an MI style or a chatbot in a CC style. Among a smaller set of participants, semistructured interviews were conducted after the chatbot interaction to gain further insights into user experience.

### Ethical Considerations

Ethics approval was obtained from the Research Ethics and Data Management Committee of the Tilburg School of Humanities and Digital Sciences (identification code REDC 2021.18ab), and the study was conducted in compliance with the ethical and data management regulations of the school. Informed consent was obtained from participants via the Qualtrics (Qualtrics International Inc) web form. Participants were recruited via the participant pools from the Tilburg School of Humanities and Digital Sciences and Tilburg School of Social and Behavioral Sciences and were compensated with credits. The study design, raw materials, and analysis plan were preregistered at the Open Science Framework.

### Participants and Procedure

Power calculations were conducted for multivariate ANOVA (MANOVA) using G*Power (version 3.1) [44], indicating that a sample size of 226 participants is adequate to uncover medium effects (effect size: ƒ^2^=0.0625; power=0.8) in accordance with previous meta-analyses on the effects of MI on smoking cessation [45]. Eligible participants were smokers (ie, had smoked at least one cigarette in the week before participation) aged ≥18 years with a competent proficiency in English reading and writing.

**Figure 1:**
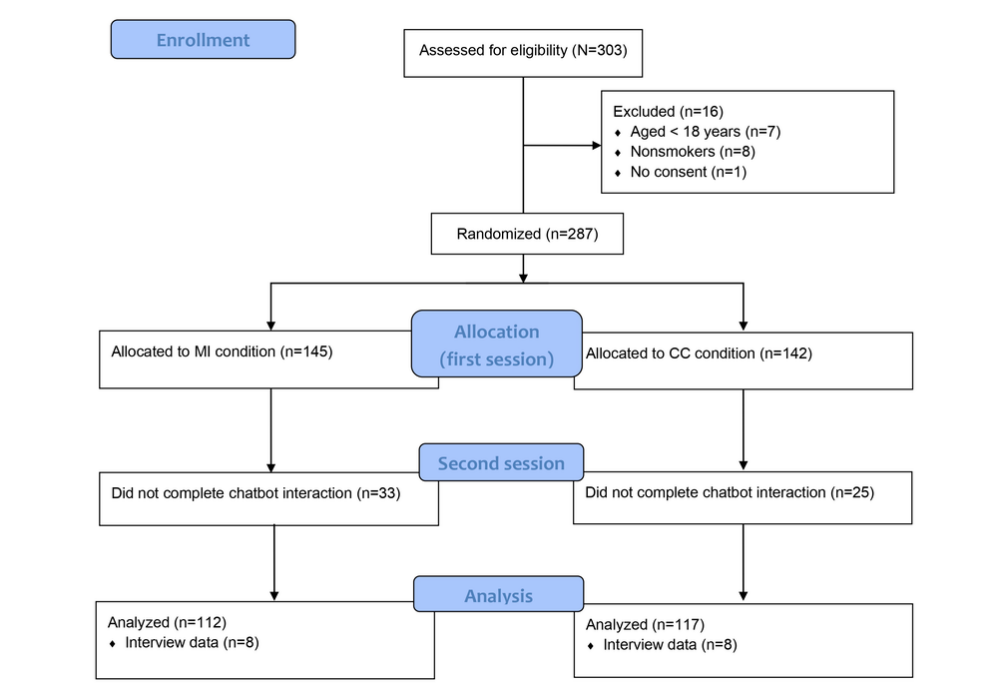
CONSORT (Consolidated Standards of Reporting Trials) flow diagram. CC: confrontational counseling; MI: motivational interviewing.

In the CONSORT (Consolidated Standards of Reporting Trials) flow diagram in Figure 1, the study procedure is visualized. Upon starting the study, participants completed the pretest questionnaire assessing demographics and baseline motivation to quit smoking, after which they were randomly assigned to interact with either the MI or the CC chatbot for 2 consecutive sessions, simulating a typical intake session and a first consultation session in smoking cessation interventions [46,47]. The randomization was double blinded and was carried out automatically by the Qualtrics software. In the first session, the chatbots carried out a conversational assessment of the participants’ smoking behavior and initial motivation and barriers to quitting smoking. After approximately 5 to 10 minutes, which resembles the waiting room setting, the second session started, in which the chatbot discussed with participants their previous quit attempts, aimed to strengthen their self-efficacy, and encouraged the participants to form future quit plans. Relevant outcome measures were assessed after each session. Upon completion, all participants were debriefed. In addition, interview invitations were sent out at random to participants in both conditions, and the final interview sample consisted of 16 participants, 8 (50%) from each condition.

### The Chatbot Interaction

#### Overview

The 2 chatbots (MI and CC) were both named Roby and were operated on the Rocket.Chat web interface. They were equipped with a natural language understanding module and a response generation module, where the former was trained using conversational data from an earlier study on an MI chatbot for smoking cessation [43]. A set of human-authored response utterances was devised by the author team and reviewed by a clinical psychology expert experienced in addiction treatment to ensure appropriateness. The chatbot scripts adhered to the Dutch national guideline for smoking cessation in primary care [48] except for the offering of pharmacological and behavioral support, in which case the chatbot referred participants to a public health website [49] for further information. The chatbot system recorded user data and conversation history to understand the “context” and selected the best-fitting utterance for the user at each turn based on the conversation context. A detailed description of the technical infrastructure is provided elsewhere [50]. The first session took approximately 5 to 7 minutes, and the second session took approximately 8 to 10 minutes.

#### The MI Chatbot Condition

The practice of MI involves a relational component and a technical component. The relational component is expressed through partnership, acceptance, compassion, and evocation [25]. The technical component includes skills such as asking open questions, reflecting on client input, affirming, summarizing, and asking for permission before providing information [14,21].

In the first session, the chatbot introduced itself and shared the agenda of the session, after which it asked about the participants’ smoking behavior and potential motivation to quit. The chatbot reflected on the participants’ input to show active attention and empathy. For example, when a participant indicated concerns about coloring teeth from smoking, the chatbot responded with the following: “You want to look and smell good, and you’re seeing that smoking might impact you on that.” The chatbot provided personalized normative feedback (ie, the percentage of smokers in their age group) after asking for permission, asked open questions, and reflected on participants’ answers. At the end of the first session, the chatbot summarized their conversation, presenting participants’ own thoughts on quitting, aiming to strengthen intrinsic motivation to quit smoking. In the second session, the chatbot asked whether the participant had made a quit attempt before and invited the participant to reflect on the experience. The chatbot discussed with participants their reasons to quit and their earlier approaches to quitting and asked the participants to think of personal strengths that helped them in their previous quit attempts. If the participant had not attempted to quit before, the chatbot asked for another challenging experience they had accomplished, aiming to elicit personal strengths and self-efficacy. Following the discussion, the chatbot summarized the conversation, let the participant review their strengths and experience, encouraged the participant to form a plan and a date for the next quit attempt, or emphasized autonomy when the participant was not ready to quit. To ensure fidelity, the chatbot dialogues were adapted from communication strategies that have been extensively tested and verified in human-delivered counseling practice [25,28].

#### The CC Chatbot Condition

Therapeutic confrontation involves a process in which the counselor provides unsolicited, direct, reality-oriented, and confrontational feedback regarding the clients’ behaviors and thoughts [51]. The main aim is for the client to realize the issues that their smoking behavior is causing. CC counselors work through identifying certain conceptions about smoking such as self-exempting beliefs [16], raising risk perception, and helping the clients realize the issue that their smoking behavior is causing.

**Figure 2:**
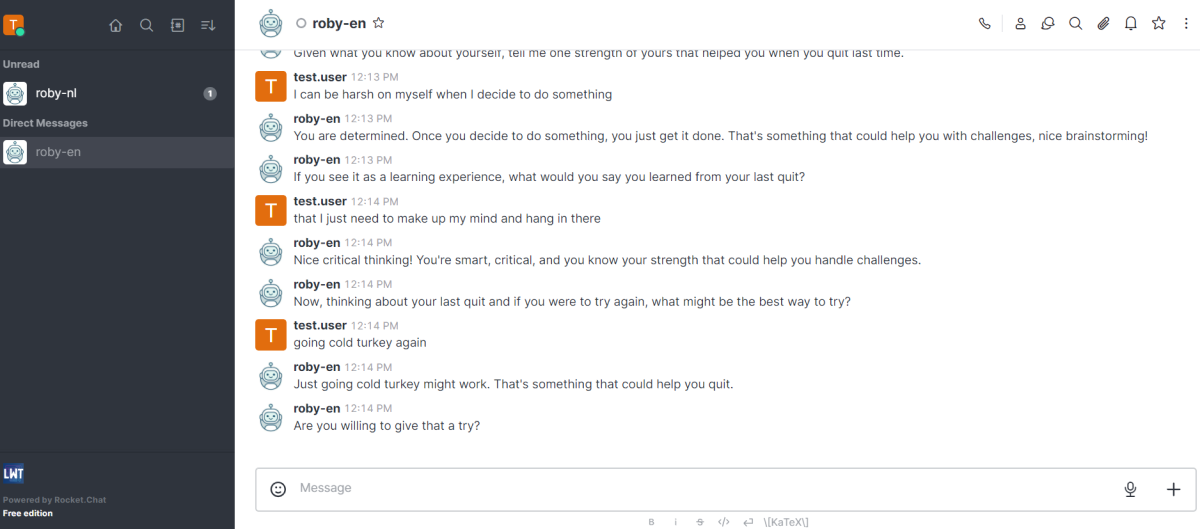
An illustration of the chat interface of the motivational interviewing condition.

In the first session, the chatbot asked about the participants’ smoking behavior while expressing concerns about their smoking severity [14,52]. While the MI chatbot elicited participants’ own motivation to quit, the CC chatbot provided factual information to confront the minimization of the issue or resistance and urge for readiness [18]. In the second session, the chatbot discussed with the participants their previous quit attempts, reminded them of their reasons to quit, and restated the risks of smoking. If the participant had not had quit attempts before, the chatbot confronted the participants’ beliefs for not quitting. An example of a typical self-exempting thought of a smoker would be the following: “I only smoke in social situations, so I don’t think it’s so bad for my health.” The chatbot then provided unsolicited factual information to challenge the smoker’s beliefs. After the discussion, the chatbot provided direct advice on quitting approach and asked for a plan and date for the next quit attempt. Except for the aforementioned manipulations, the content of the dialogues (eg, questions, topics, and information provided) and the length of the interaction were as similar as possible across the conditions. Figure 2 shows an example of the final conversation. The complete chatbot scripts can be found in the Open Science Framework preregistration.

### Measures

Demographics and smoking-related baseline information were collected at the pretest time point. Age and gender were assessed using single items. Baseline motivation to quit was measured using the Contemplation Ladder [53], where participants indicated their motivational status ranging from 0 (*no thoughts on quitting*) to 10 (*taking action to quit*). Level of nicotine dependence was measured using the Fagerström Test for Nicotine Dependence [54], and average daily cigarette consumption was measured using a single item.

For outcome measures, we assessed both cessation-related variables and user experience–related variables, and all variables were measured both after the first session (T1) and after the second session (T2). All scale variables were measured using 5-point scales. The measurement scheme followed that of the earlier study [43] to facilitate comparison.

Intention to quit was measured using the Contemplation Ladder [53]. Participants indicated where they identified themselves on a single item, with responses ranging from 0 (*no thoughts on quitting*) to 10 (*taking action to quit*).

Self-efficacy was assessed using the Smoking Self-Efficacy Questionnaire [55]. On a 5-point scale, participants indicated whether they thought they would be able to refrain from smoking in various difficult situations. An example question is as follows: “how sure are you that you could refrain from smoking when you feel nervous.”

Engagement with the chatbot interaction was assessed using 9 items from subscales of the short form of the User Engagement Scale [56]. The esthetic appeal subscale was removed as this study focused on the communication process instead of the interface design. An example item is as follows: “I was absorbed in this experience.” An additional question was included asking about participants’ endorsement for future use.

Therapeutic alliance was measured using the Working Alliance Inventory–Short Revised [57], a 12-item self-report measure used to assess the relationship between participants and the chatbot. An example item is as follows: “We agree on what is important for me to work on.”

Perceived empathy was measured using a 3-item (eg, Roby seemed to understand me) scale based on research on interpersonal communication by Rubin and Martin [58].

Interaction satisfaction was measured using 5 items on a 5-point scale ranging from “Not at all” to “Very much” following studies on telephone smoking cessation counseling [59]. An example item is as follows: “how was your counsellor in terms of being a good listener?”

As a manipulation check question, perception of MI was measured using an 8-item scale based on the Client Evaluation of Motivational Interviewing scale [60]. Example items are “Roby argued with you to change your behavior” and “Roby helped you feel confident in your ability to change your behavior.”

### Statistical Analysis

Experiment data were analyzed using R (version 4.2.1; R Foundation for Statistical Computing). Independent-sample 2-tailed *t* tests and a chi-square test were conducted to check for equal distribution of background variables (ie, gender, age, nicotine dependence level, and baseline motivation to quit) across conditions. Variables that were not equally distributed across conditions were included as covariates in hypothesis testing.

To test the preregistered hypothesis regarding the effect of MI chatbot conversations on the outcomes, a 1-way MANOVA was performed using the 6 outcome variables (ie, self-efficacy, intention to quit, engagement, therapeutic alliance, perceived empathy, and interaction satisfaction). Data measured at T2 were used for this hypothesis.

### Qualitative Data Collection and Analysis

Qualitative data were gathered at the postmeasurement time point through semistructured video interviews with 16 participants, 8 (50%) each randomly selected from the 2 conditions. The interviews lasted 20 to 30 minutes each and were conducted by the first author, who was familiar with the chatbot content. Before study commencement, an interview guide was developed by the author team and pilot-tested. The interview topics were guided by the theoretical framework of acceptability of health care interventions [61], which consists of several constructs (eg, affective attitude, burden, intervention coherence, and perceived effectiveness) that closely relate to user experience with health care interventions.

The interviews were audio recorded and transcribed. The transcripts were analyzed using thematic analysis [62]. We adopted a hybrid approach combining deductive and inductive coding [63] in which coding was mainly informed by themes that could potentially answer the research questions and inform chatbot improvement but also allowed for novel codes to emerge for additional insights. A pilot coding round was carried out by 2 independent reviewers, and consensus regarding the codes was reached through discussion. Coding was performed in an iterative process over multiple reads of the transcripts. Multiple group meetings were organized aiming to identify potential biases in the coding and interpretation process. One author (LH) carried out the coding of the remaining interviews. The analysis procedure was performed using the ATLAS.ti (ATLAS.ti Scientific Software Development GmbH) program.

## Results

### Quantitative Results

#### Sample Characteristics and Descriptive Results

A total of 303 participants were recruited, with 287 (94.7%) being eligible for participation. Among the eligible participants, 50.5% (145/287) were allocated to interact with the MI chatbot, 49.5% (142/287) were allocated to the CC chatbot, and 20.2% (58/287) were excluded from the analysis for not completing the chatbot interaction and questionnaire, resulting in a final analyzed sample of 229 participants. Of these 229 participants, 153 (66.8%) were female, and the average age of the participants was 21.1 (SD 2.75) years. The average Fagerström Test for Nicotine Dependence score was 1.17 (SD 1.71), indicating overall low dependence on nicotine. The 2 groups did not differ significantly on any of the background variables at the pretreatment time point. Table 1 shows the sample characteristics and the *P* values.

All measures used demonstrated high reliability. Table 2 shows the reliability and descriptive results for the outcome variables.

**Table 1:** Demographic and tobacco use characteristics of participants at baseline by condition (n=229).

Characteristic			Total		MI^a^ (n=112)		CC^b^ (n=117)		*P* value	
Age (y), mean (SD)			21.06 (2.75)		21.17 (3.04)		20.95 (2.44)		.55	
**Sex** **, n (%)**										.51
	Female	153 (66.8)		71 (63.4)		82 (70.1)				
	Male	73 (31.9)		39 (34.8)		34 (29.1)				
	Other	3 (1.3)		2 (1.8)		1 (0.9)				
Daily cigarette consumption, mean (SD)			4.90 (5.01)		5.06 (4.95)		4.74 (5.08)		.64	
FTND^c^ score, mean (SD)			1.17 (1.71)		1.19 (1.73)		1.15 (1.71)		.88	
Score of baseline motivation to quit, mean (SD)			5.33 (2.82)		5.31 (2.84)		5.34 (2.81)		.94	

^a^MI: motivational interviewing.

^b^CC: confrontational counseling.

^c^FTND: Fagerström Test for Nicotine Dependence.

**Table 2:** Descriptive and reliability results of the outcomes.

	T1 (first session)			T2 (second session)		
	MI^a^, mean (SD)	CC^b^, mean (SD)	Cronbach α	MI, mean (SD)	CC, mean (SD)	Cronbach α
Intention to quit	5.71 (2.80)	5.69 (2.81)	—^c^	6.26 (2.85)	5.91 (2.85)	—
Self-efficacy	2.62 (0.76)	2.79 (0.88)	0.85	2.74 (0.76)	2.82 (0.92)	0.87
Engagement	3.41 (0.67)	3.38 (0.66)	0.83	3.40 (0.73)	3.14 (0.77)	0.87
Therapeutic alliance	3.47 (0.65)	3.18 (0.75)	0.90	3.61 (0.67)	3.22 (0.85)	0.92
Perceived empathy	3.51 (0.84)	3.00 (0.92)	0.81	3.59 (0.92)	2.94 (1.10)	0.91
Interaction satisfaction	3.81 (0.67)	3.50 (0.77)	0.73	3.85 (0.78)	3.41 (0.80)	0.78

^a^MI: motivational interviewing.

^b^CC: confrontational counseling.

^c^Not applicable.

#### Main Effects of MI

We hypothesized that the MI-style chatbot would lead to better outcomes in terms of both motivating cessation and user experience. We performed a manipulation check on participants’ perception of MI using the Client Evaluation of Motivational Interviewing scale [60], which showed that participants in the MI condition perceived the chatbot as significantly more MI-like (mean score 3.77, SD 0.62) than participants in the CC condition (mean score 3.21, SD 0.60; t_227_=6.89; *P*<.001), representing a large effect size (*d*=0.91). Thus, the manipulation was deemed successful. For this preregistered hypothesis, the 2 sessions were regarded as 1 interaction, and we used data measured after the entire interaction (ie, at T2). Results of a subsequent MANOVA using T2 data revealed significant overall differences between the 2 groups (Pillai trace=0.12; *F*_6,222_=5.00; *P*<.001; η*^2^*=0.12). We performed a follow-up discriminant analysis to examine which outcomes contributed the most to the overall group differences [64]. Results showed that user experience–related outcomes (ie, engagement, perceived empathy, and interaction satisfaction) had the highest correlation coefficients with the discriminant function, indicating that they mostly differentiated between the MI and CC conditions, whereas cessation-related outcomes (ie, intention to quit and self-efficacy) practically did not differ between the 2 groups. These results are presented in Table 3. Overall, the results of the MANOVA and discriminant analysis suggest that chatbot counseling style (MI vs CC) has a significant effect on the outcome variables and that the effect can be mostly explained by differences in user experience.

**Table 3:** Results of discriminant function analysis of the outcome variables by condition.

Variable	Discriminant function	
	Correlation coefficients with discriminant function	Standardized coefficient with discriminant function
Intention to quit	0.00	0.01
Self-efficacy	–0.19	–0.16
Engagement	–*0.80*^a^	–*0.60*
Therapeutic alliance	0.23	0.18
Perceived empathy	*0.69*	*0.71*
Interaction satisfaction	*0.85*	*0.67*

^a^Italics indicate variables that mostly discriminated the 2 conditions.

#### Exploratory Analyses

**Figure 3:**
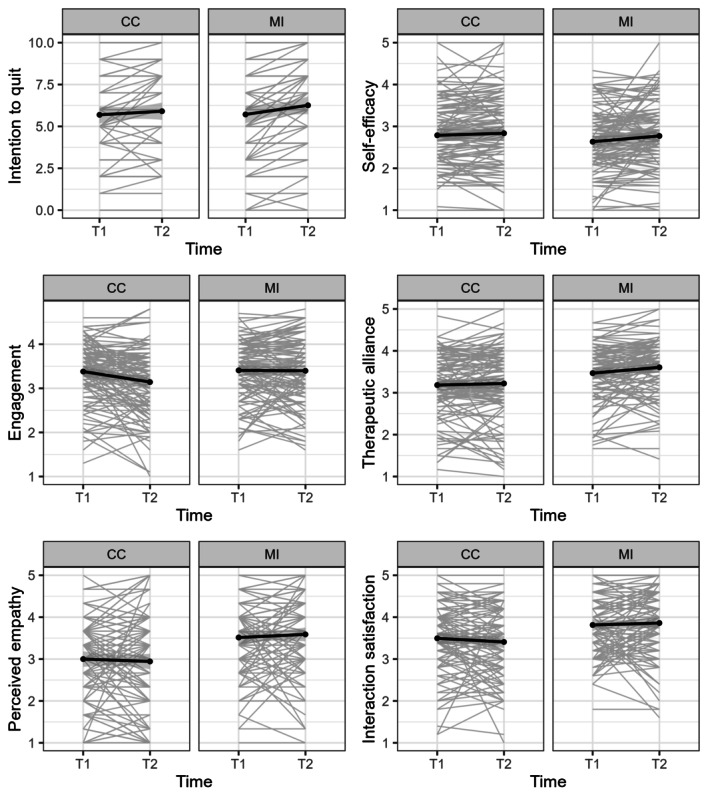
Changes over time in individual tracings and group means for the outcome variables. CC: confrontational counseling; MI: motivational interviewing.

As the chatbot interaction involved 2 sessions, we explored potential changes in participants’ experience over the 2 sessions (ie, the multi-session effect). We performed a mixed ANOVA for each of the outcome variables, including condition (MI vs CC) as a factor and time (T1 vs T2) as a repeated measure. Test statistics can be found in Table 4, and means and SDs can be found in Table 2. For intention to quit, there was a significant effect of time (*F*_1,227_=39.02; *P*<.001). Participants’ intention to quit increased after the interaction, and such increase was significantly more profound in the MI condition (*F*_1,227_=7.43; *P*=.007). For *self-efficacy*, an increase was found over time (*F*_1,227_=6.89; *P*=.009), and the increase was only significant in the MI condition (*F*_1,227_=4.34; *P*=.04). For *engagement*, participants had a significant overall decrease in their engagement with the chatbot (*F*_1,227_=10.07; *P*=.002). A significant interaction effect between time and condition was also found, and follow-up simple-effect analysis per condition showed that the CC condition significantly contributed to the overall decrease (*F*_1,227_=8.55; *P*=.004). For the remaining 3 user experience–related outcomes, the MI chatbot was rated significantly better in terms of *therapeutic alliance* (*F*_1,227_=13.61; *P*<.001), *perceived empathy* (*F*_1,227_=25.90; *P*<.001), and *interaction satisfaction* (*F*_1,227_=18.14; *P*<.001). Finally, a significant overall increase over time in therapeutic alliance was found in both conditions (*F*_1,227_=6.64; *P*=.01). The results are visualized in Figure 3.

**Table 4:** Results of repeated-measure ANOVA on the outcomes.

	Condition			Time			Condition × time^a^			Simple-effect post hoc analysis of the interaction effect				
										Effect of time in MI^b^			Effect of time in CC^c^	
	*F* test (*df*)	*P* value	*F* test (*df*)		*P* value	*F* test (*df*)		*P* value	*F* test (*df*)		*P* value^d^	*F* test (*df*)		*P* value
Intention to quit	0.26 (1, 227)	.61	39.02 (1, 227)		<.001	7.43 (1, 227)		.007	31.30 (1, 111)		<.001	8.41 (1, 116)		.008
Self-efficacy	1.14 (1, 227)	.29	6.89 (1, 227)		.009	4.34 (1, 227)		.04	8.03 (1, 111)		.01	0.23 (1, 116)		>.99
Engagement	2.74 (1, 227)	.10	10.07 (1, 227)		.002	8.55 (1, 227)		.004	0.03 (1, 111)		>.99	17.2 (1, 116)		<.001
Therapeutic alliance	13.61 (1, 227)	<.001	6.64 (1, 227)		.01	2.12 (1, 227)		.15	—^e^		—	—		—
Perceived empathy	25.90 (1, 227)	<.001	0.03 (1, 227)		.87	1.54 (1, 227)		.22	—		—	—		—
Interaction satisfaction	18.14 (1, 227)	<.001	0.24 (1, 227)		.63	2.28 (1, 227)		.13	—		—	—		—

^a^The interaction effect between time and condition.

^b^MI: motivational interviewing.

^c^CC: confrontational counseling.

^d^Bonferroni-adjusted *P* value.

^e^Not applicable.

### Qualitative Results

#### Overview

The qualitative data provided insights into the user experience with the chatbots, participants’ perceptions of the motivating aspect of the chatbots, and suggestions for future design. Moreover, the results supported and expanded on the experimental comparison between the MI- and CC-style chatbots. The findings centered on 3 main themes with accompanying subthemes. The themes were *prior expectation*, with the subthemes *authoritative confrontation* and *generic interaction*; *timely experience*, with the subthemes *affective attitude*, *engagement*, and *usability*; and *postinteraction reflection*, with the subthemes *perceived effectiveness*, *perception of health chatbots*, and *ideal intervention*. Table 5 presents the themes and illustrative quotes.

**Table 5:** Illustrative quotes for the qualitative findings.

Main theme and subtheme			Participant quotes
**Prior expectation**			
	Authoritative confrontation	“I have the expectation that it was going to be some sort of, gaslighting situation, you need to stop smoking because this and this.” [Participant 5; female, aged 19 years; MIa condition]	
	Generic interaction	“I thought it was just going to be generic for everybody the same, not regarding the answers the person gave. I didn’t think it was going to be personalized.” [Participant 2; female, aged 22 years; CC^b^ condition]	
**Timely experience**			
	Affective attitude	“I had fun chatting with the chatbot. And in my opinion, the chatbot was really friendly and understanding.” [Participant 10; female, aged 18 years; CC condition] “It didn’t feel like I was being put into a position where I can feel guilty about my smoking habits. He actually made sure that I felt safe.” [Participant 5; female, aged 19 years; CC condition]	
	Engagement	“In the conversation, I lost my engagement for a moment in the situation with finding correct answers that the chatbot will understand.” [Participant 7; female, aged 24 years; MI condition] “It was just enough to keep the conversation going but not extremely involved.” [Participant 3; female, aged 19 years; CC condition] “I found it really interesting because I couldn’t foresee what this conversation would bring to me, so it’s always nice to keep engaged in the conversation and see what will come out of it.” [Participant 10; female, aged 18 years; CC condition] “I knew it was a chatbot and everything, so if it was a person, I would be more engaged.” [Participant 2; female, aged 22 years; CC condition]	
	Usability	“It’s easier to make a chatbot than to find a therapist who would guide you through the process, and I also think it’s good to have a computer because you can always text him at any time of the day.” [Participant 13; female, aged 19 years; MI condition] “The amount of information was a bit, not really enough, I feel like it could have given me more information about certain things.” [Participant 3; female, aged 19 years; CC condition]	
**Postinteraction reflection**			
	Perceived effectiveness	“I really like the question ‘what did you feel when you stopped smoking?’ I really asked myself how I felt, why did I stop. And it was really good, looking at that time, it was good for me to see that I felt good, maybe I should do it again.” [Participant 13; female, aged 19 years; MI condition] “I’m actively thinking about it [quitting] and actively attempting at sometimes, but it didn’t bring out any new techniques or methods or reasons to.” [Participant 4; male, aged 29 years; MI condition]	
	Perception of health chatbots	“As long as I know that it would be destroyed after it’s not needed anymore, I’d be fine with it. I think maybe it’s even easier to tell your feelings to a bot, which you know is programmed to not be judgmental and just want to help, and also it’ll be gone.” [Participant 9; male, aged 21 years; CC condition] “It’s quite scary because technology is evolving so much that you could actually have a conversation with the bot about something so subjective and personal.” [Participant 11; female, aged 18 years; MI condition] “I wasn’t fully comfortable because like I said in the back of my mind it was a computer, and I thought it was not going to understand my emotions, so I didn’t really feel like telling them.” [Participant 13; female, aged 19 years; MI condition]	
	Ideal intervention	“I’m interested in people’s thoughts and why they do things, like what’s the inner reason for them to do that. Because it’s the most effective way to find a solution. Just need the why’s. If a bot could that, it would be better.” [Participant 6; female, aged 21 years; CC condition] “Because you gave the bot all that information and it’s stored somewhere, you can also use that information to make [progress] charts available for the user.” [Participant 4; male, aged 29 years; MI condition]	

^a^MI: motivational interviewing.

^b^CC: confrontational counseling.

#### Theme 1: Prior Expectation

Almost all participants had some expectations regarding the chatbot interaction when they signed up for participation. Most people referred to their previous experience with other chatbots as baseline expectations and expected nonpersonalized *generic interaction*. Customer service chatbots were the most frequently mentioned comparison, and participants were expecting similar general task-oriented conversations. One participant mentioned the following:

I just thought it would be a conversation the same as when you text customer service or something. And then they have a few answers there and they send it to you no matter what question you asked.

Participant 12; female, aged 20 years; MI condition

In terms of conversation content, many participants expected to passively receive persuasive information, such as risks of smoking and benefits of quitting. The chatbot was imagined as an *authoritative* figure in a formal setting (eg, a physician) giving advice. Most people expressed positive expectations regarding factual information and guidance, which could be motivating to quit. A few other participants mentioned that the anticipated confrontation from an authoritative figure could be intimidating and induce resistance at the beginning. Most participants later noted that the chatbot’s nonjudgmental, supportive, and personalized approach was much better than expected. One participant put it as follows:

I was kind of, I wouldn’t say scared, but I wasn’t really willing to start the conversation because I was like “Oh, this is going to be, you have to quit, you should not smoke.” But then I was surprised because it wasn’t like that.

Participant 1; female, aged 25 years; CC condition

#### Theme 2: Timely Experience

The second theme was related to people’s timely experience during the chatbot interaction. Participants had an overall positive *affective attitude* toward the interaction, and it was largely driven by the hedonic and therapeutic experience. Almost all participants found the chatbot interaction fun and enjoyable, attributing it to the friendly and knowledgeable feeling of the chatbot. Participants appreciated the safe space that the chatbot created for them to freely discuss their smoking behavior without being judged. Many participants expressed that they felt respected and supported by the chatbot, and they acknowledged that such a safe space is important for counseling and reducing resistance. However, a small number of participants from the CC condition mentioned that they felt coerced and confronted. One participant mentioned the following (more illustrative quotes can be found in Table 5):

He was actually pretty understanding, he wasn’t judging. And I think that was the best part of it because I know what it’s like to be judged by people and it felt really good not to. He was actually being helpful without being judgmental.

Participant 5; female, aged 19 years; MI condition

*Engagement* with the interaction was frequently discussed by participants both positively and negatively and was largely driven by people’s technical experience. When the interaction went smoothly without many technical errors (eg, chatbot misunderstanding and asking for rephrasing), people felt engaged with the interaction, and disengagement occurred when the conversation was interrupted by technical glitches. Despite being engaged in the conversation, a few participants mentioned that their engagement level was limited to task completion (ie, completing their research participation) and they were not emotionally involved. Supporting the experimental findings, more interviewees in the MI condition mentioned that they felt engaged with the chatbot compared to participants in the CC condition. In addition, some people compared chatbot interaction with human-human interaction and generally preferred engaging with humans for more flexibility and true understanding. One participant stated the following:

A human can understand whatever you say, a chatbot sometimes has issues processing information. It’s just for me, I’m more emotionally involved when talking to a person than talking to a computer.

Participant 13; female, aged 19 years; MI condition

In terms of *usability*, almost all participants felt that the chatbot was easy to use, and some participants extrapolated such comments to health chatbots in general, acknowledging the highly accessible benefit of chatbots. While some participants thought that the chatbot provided the right amount of information, which was easy to process and not overwhelming, more people felt that the amount of information was not sufficient to motivate quitting in and of itself, and they emphasized that more less known information is needed. One participant suggested the following:

The fact that most people don’t smoke and that 1/3 of smokers decided to quit was a new fact for me, and it affected me. But I needed some more facts, so the quantity is, I think, not enough. It could give more things.

Participant 6; female, aged 21 years; CC condition

#### Theme 3: Postinteraction Reflection

The third theme captured people’s reflection on how the chatbot influenced their motivation and suggestions for improvement. Most participants discussed their *perceived effectiveness* of the chatbot interaction in a positive way, which parallels the experiment finding that both chatbots led to an increased intention to quit. Many people acknowledged that the conversation encouraged them to reflect on their behavior and brought them new perspectives on their thoughts about smoking. Novel factual information (eg, number of smokers at a certain age) was another highly appreciated component of the interaction, identified as a motivating factor by almost all participants. However, a few people explicitly expressed that the chatbot did not motivate them. Part of this ineffectiveness stemmed from participants’ low readiness to quit, whereas a number of people perceived the conversation as reiterating already known information (eg, health benefits of quitting). Substantial differences were found between interviewees from the 2 experimental conditions such that it was mentioned more in the MI condition that people appreciated the chatbot encouraging self-reflection and more participants in the CC condition found the factual information useful:

I think it touched my soul a little bit. I tried to think about my habits and what I don’t like. And I think that considering what I don’t like helped me the most to realize even more why I should quit.

Participant 7; female, aged 24 years; MI condition

When discussing their *perception of health chatbots*, most participants felt comfortable talking about smoking and health-related topics in general with the chatbot. The chatbot was perceived as an accessible conversational partner that is nonjudgmental with helping intentions. Several participants compared chatbots with humans and noted that chatbots are particularly useful because they require less logistic and mental effort. However, a number of participants also made a clear distinction between chatbots and humans, and they found it hard to anthropomorphize them. They felt that the chatbot conversation has a fixed flow and chatbots have no real emotional capability, which differentiated them from real humans. While many people reported being aware of and appreciated the anonymous nature of the interaction and the temporary storage of data, a few participants expressed concerns about technology in general. They felt uncanny that technology was advancing at a speed that it could analyze human emotions and were worried that such technology was premature in terms of true understanding. In addition, there was a perception of risk in data sharing and privacy. For example, one participant commented the following:

If I were to talk to a human counselor, they are bounded by professional secrecy, and the bot, even though it is used by a professional and that professional is bounded to secrecy, the bot’s data is stored somewhere. Data leaks happen all the time.

Participant 4; male, aged 29 years; MI condition

Finally, participants discussed the *ideal intervention* they would like for smoking cessation. The most frequently mentioned suggestion for intervention content was that more background stories should be explored (eg, why one started smoking and what is the triggering situation for one to smoke) to increase personal relevance. Many participants also mentioned that they would like to discuss quitting methods in more detail to feel confident. Specifically, regarding the use of chatbots, most participants preferred them as a standby tool that can respond to users’ questions and provide timely advice. Users would like to be in contact with the chatbot when lacking motivation or having cravings and would like to receive practical tips from the chatbot. In terms of long-term use, several participants suggested that the chatbot could generate progress reports and have adjusted conversations at different progress points.

## Discussion

### Principal Findings

In this mixed methods study, we aimed to investigate the effectiveness and user experience of MI strategies delivered by a chatbot. Results showed support for our hypothesis regarding the advantage of the MI chatbot in terms of user experience (ie, engagement, therapeutic alliance, perceived empathy, and interaction satisfaction), but no significant effect was found for the cessation-related outcomes (ie, intention to quit and self-efficacy). Importantly, exploratory analyses showed increases in participants’ intention to quit, self-efficacy, and therapeutic alliance over the sessions, suggesting the usefulness of chatbots for this purpose. These increases were more profound in the MI condition (except for therapeutic alliance, for which the increase over time did not differ between conditions), suggesting the potential of MI in the long run. Finally, participants reported an overall decrease in their engagement with the chatbot. Qualitative data supported and expanded the experiment findings. We identified constructs that relate to users’ prospective acceptability, timely experience, and retrospective acceptability of our smoking cessation chatbot, with several insights applicable to health chatbots in general.

Our findings regarding user experience are in line with those of previous research not involving chatbots demonstrating clients’ preferences for an empathetic and collaborative approach. Previous research in health counseling has highlighted patients’ preferences regarding an empathic and collaborative approach [14,18], and our findings echo such preferences and extend their value to the chatbot setting. Our qualitative results provided further possible explanations for this. Participants in the MI condition mentioned more frequently than participants in the CC condition that they felt understood by the chatbot and that they were more engaged in the conversation. In the CC condition, participants more frequently felt coerced and confronted by the chatbot, and they indicated that they would like more personal conversation and that the provided information was not sufficient. These findings show that participants value the relational aspect of the chatbot, suggesting that it is essential for the chatbot to have a person-centered approach and have a supportive attitude. Moreover, several participants mentioned having previous expectations of an authoritative and confrontational figure sending generic information, whereas the MI chatbot exceeded their expectations with its personalized, understanding, and empathetic approach. Client expectations regarding treatment are known as a key factor in counseling outcomes and satisfaction [65]. Expectancy violation theory suggests that positive expectation violation (eg, the chatbot was more personalized than expected) predicts more favorable outcomes than negative expectation confirmation (eg, the chatbot was confrontational as expected) [66]. Indeed, despite recent research favoring MI, many addiction counselors may still use confrontational techniques. Additionally, smokers often face criticism from their social environment, which may influence their expectations of how a chatbot will interact with them [16,51]. In other words, the previous expectation of a CC-like chatbot interaction may account for the positive user experience with the MI chatbot.

In terms of the motivating effect on smokers’ intention to quit and self-efficacy, our results did not demonstrate significant differences between the 2 chatbot styles. The MI chatbot did not evoke more motivation or self-efficacy than the CC chatbot. One plausible explanation for why we did not find differences regarding the different styles could be that our participants were recruited with established motivation at baseline. On average, participants in both conditions placed themselves on the baseline contemplation ladder between “I think I should quit but not quite ready” and “I am starting to think about how to change my smoking patterns.” Smokers at this stage of change might appreciate both encouragement and concrete guidance on quitting [67]. In other words, participants may value the intrinsic motivation-evoking aspect of MI, whereas the direct advice in CC is perceived as useful at the same time. Together, these effects may have resulted in nonsignificant differences in motivating intention to quit between the 2 styles. Another interpretation stems from the particular chatbot setting. While we hypothesized the MI chatbot to be helpful in evoking motivation and encouraging personally achievable quit plans, its effectiveness might depend on clients’ actual perception of it. The fidelity of MI requires true understanding and empathy, and it remains unclear whether the chatbot encompasses these qualities. Research in human-computer interaction has suggested that inaccurate artificial empathy might be less favorable than no empathy [68,69]. This is supported by our interview findings such that, despite the clearly appreciated nonjudgmental and supportive tone of the MI chatbot, people preferred discussing emotional experiences with real humans because chatbots do not have emotional capacities. On the other hand, while CC was expected to have less positive effects, using a chatbot as the portrayed counselor might have mitigated the anticipated resistance. As noted by both our interview participants and previous research, health chatbots are predominantly perceived as an instrumental tool that answers questions and provides information [70], and therefore, people might value the informational aspect more than the therapeutic aspect. However, it is important to highlight that, regardless of the condition, participants overall reported a small yet significant increase in their motivation to quit and self-efficacy after the chatbot interaction. This is consistent with previous research suggesting that minimal conversation about smoking cessation can affect quitting intentions and behaviors [71], and chatbots might be particularly useful due to their high accessibility. It is conceivable that the use of a chatbot alone is effective, and the specific communication strategies used may not have a major impact on the outcomes [43]. To better realize their potential, future research is needed to understand the impact of different automatic conversation strategies and the role of users’ communication preferences regarding chatbots and design chatbots that combine effective strategies from different counseling approaches.

Another notable finding is that people’s engagement with the chatbot decreased over time. Despite the fact that our experimental setting consisted of only 2 sessions, this provides a stepping stone for the long-term use of chatbots, which is particularly important for smoking cessation, which requires sustained effort. Research in human-computer interaction shows that user engagement tends to decline over time [72-74]. In addition to the novelty effect (ie, people engage with new technology due to curiosity, which declines gradually [75]) that has been proposed to explain declining engagement [12], our study found that users’ technical experience was an active determinant in the engagement process. The most frequently mentioned reason for participants to disengage from the chatbot interaction was the encountered technical errors (eg, chatbot misunderstanding and repairing). Being able to input free text was one of the aspects that participants found engaging. This provides a plausible explanation for the finding that the MI chatbot was perceived as more engaging than the CC chatbot such that the open-ended questions asked by the MI chatbot offered users active involvement in the conversation. To increase people’s engagement with the chatbot and the intervention, future research is needed to identify more factors that influence the engagement process. One area worthy of investigation relates to the automatic detection of engagement or disengagement from user utterances so that the chatbot can respond in time to keep the users engaged. For example, He et al [76] explored textual cues indicative of user engagement and suggested using cognitive strategies (eg, quizzes and reflective questions) to keep the user engaged.

### Limitations

A number of methodological limitations should be borne in mind when interpreting the results. First, the length of the interaction was fairly short. We aimed to simulate a brief intake session and a first counseling session, and our results were in line with research on the positive effects of brief interventions for smoking cessation [77]. Even though we found a significant increase in smokers’ motivation and self-efficacy to quit, this increase was relatively small, which is not unexpected given the briefness of the interaction. Our exploratory results suggest that the small but positive effects increased over time. The promising outlook calls for studies that involve prolonged use to seek more meaningful changes and investigate whether the effects can be sustained in the longer term. We believe that chatbots are well suited for addressing this long-term objective as they facilitate extended and personalized interactions, and we plan to delve deeper into this potential in a follow-up longitudinal study. In addition, we measured intention rather than actual cessation as a primary outcome, which provided initial support for the usefulness of the chatbot interaction. To further this line of research, behavioral outcomes are needed to ascertain the effectiveness of chatbot interventions. Moreover, objective use data (eg, interaction length and sentence length) can be used to complement self-reported engagement. It is recommended that future research include more objective instruments such as biochemical and behavioral measures to validate the effects of chatbot interaction. In addition, although our sample represents the main user group of new technologies such as chatbots, their smoking behaviors might not be representative of the general smoking population. Their familiarity with technology might have also led to results that are less applicable to other user groups. Considering the potential impact of demographics and smoking behaviors, it is important to design chatbots that can cater to a broader range of populations.

### Implications for Future Research and Practice

Our findings, coupled with previous research, suggest that chatbots have the potential to motivate smokers to quit, and equipping them with MI skills could improve user experience. However, the insignificant results on cessation-related outcomes call for more research to further identify strategies that can effectively motivate cessation. For example, previous research has attempted to understand the working mechanism of MI in an automated setting by comparing different types of open-ended questions and reflections and suggested the advantage of incorporating multiple-choice questions particularly in an automated setting, expanding on the general guideline of using open-ended questions in traditional MI [28,78]. Therefore, it is important to disentangle the MI components and translate active ingredients to a chatbot setting. Moreover, our findings suggest that the directive informational aspect of the CC chatbot was appreciated by the participants, and future research is encouraged to combine such aspects with the nonjudgmental spirit of MI to capture the best of both worlds. In addition, in view of the person-centered approach and the potential of personalization, it is important to explore individual factors that can influence people’s preferences and experience with the chatbots. For instance, previous research has found that users’ need for autonomy and their self-efficacy was influential in their responses to an MI or a CC chatbot [79], highlighting the importance of addressing users’ unique needs in designing future chatbot interventions. As noted in the qualitative findings, technology-delivered counseling has both positive (eg, easy access and facilitated disclosure) and negative (eg, lack of true understanding) effects. Considering the interpersonal nature of health counseling, it is essential to understand the role of the human touch in chatbot-delivered interventions. Our study provided some initial insights into this, such as that the chatbots facilitated health-related disclosure but people are skeptical of their ability to understand human feelings. Future work is needed to further understand how people perceive chatbots in an interpersonal setting; for example, how do people view the relational communication delivered by a chatbot? How do people disclose their emotions and understand chatbots’ responses? This line of research will help improve the design of chatbot-delivered MI and health counseling in general.

On a practical note, researchers and practitioners should be cautious in humanizing chatbots. Even though research has pointed to the potential of empathetic agents [40], overly imitating human emotions could result in an uncanny feeling [80]. Our findings suggest that, while humanlikeness was generally favored, participants felt hesitant discussing emotions with the chatbot when they were aware of its mindless nature. To best equip chatbots with social cues, future work needs to understand users’ perceptions of different facets of emotions. For example, cognitive empathy (ie, understanding others’ perspectives and emotions) from a chatbot is appreciated, whereas affective empathy (ie, emotional response to others’ emotions) induces perceived eeriness [38]. Moreover, we argue that chatbots should be considered a supplementary tool rather than a replacement for human caregivers, especially in health-related contexts where inaccurate and invalid information can have a harmful impact on people’s well-being. Several of our interview participants suggested that the chatbot should be a standby conversational tool that provides timely support. Functions supporting long-term use (eg, quitting progress report) should be considered as well.

An important implication of this study is that previous expectations play a crucial role in people’s experiences with and responses to chatbot interventions. While people used to have expectations of a customer service–like generic chatbot, such expectations might be drastically changed by recent rapid technological advancements. In particular, ChatGPT, one of the latest large language models, has captured the attention of millions since its release. It might reshape people’s expectations of chatbots with its large amount of knowledge and natural text generation and its capability to respond to any conceivable input. However, it should be made clear to the public that such technologies should not be regarded as a replacement for medical authorities and are not ideal for long-term support as it requires personalized care and tailored interventions, whereas ChatGPT’s capabilities are limited to the general information that it was trained on. In addition, it is well established that chatbots relying on large language models may give socially desirable answers, may suffer from undesired biases, and are not necessarily truthful [81]. Despite the extensive amount of information available, such technologies simply answer to requests and often overlook user background and conversational contexts. We are aiming to develop chatbots that can understand and engage with users in the long term while ensuring accuracy and safety. Health care chatbots need to balance safety measures (eg, using prescripted utterances to avoid harmful content) with engagement features (eg, flexibility and variety in texts). Technologies such as ChatGPT could bring additional support by providing coherently written information, but human control should be involved to ensure safe, responsible, and accountable use.

### Conclusions

This study adds to the body of research regarding the effectiveness and user experience of chatbots using 2 different counseling styles. The results point in the direction that chatbots are a promising tool in motivating cessation and the use of MI can improve user experience. Several constructs such as affective attitude and engagement were identified to understand users’ previous expectations, timely experience, and retrospective acceptability of the chatbot interaction. These findings highlight the potential of chatbots for smoking cessation and suggest a few avenues for future research.

## Abbreviations

CC: confrontational counseling
CONSORT: Consolidated Standards of Reporting Trials
MANOVA: multivariate ANOVA
MI: motivational interviewing
